# Supplementary material for: People infer communicative action through an expectation for efficient communication
Source: Nat Commun. 2022 Jul 18;13:4160. doi: 10.1038/s41467-022-31716-3 (PMC9293910; doi:10.1038/s41467-022-31716-3)
Supplement: Supplementary file 1 — Supplementary Information [file 41467_2022_31716_MOESM1_ESM.pdf]

Supplementary Information

for *People infer communicative action through an expectation for efficient communication*

Amanda Royka[1], Annie Chen[2], Rosie Aboody[1], Tomas Huanca[3], Julian Jara-Ettinger[1,2,4]

[1] Department of Psychology, Yale University

[2] Department of Computer Science, Yale University

[3] Centro Boliviano de Desarrollo Socio-Integral

[4] Wu Tsai Institute, Yale University

1 Computational model

Our model-based analyses are based on a family of computational models of instrumental goal inference known broadly as *inverse planning* [1, 2, 3, 4, 6]. These models are typically take as input an environment, a set of potential instrumental goals, and a set of actions. Using the assumption that agents act efficiently in space, these models then identify the instrumental goal that the agent is pursuing by computing the posterior distribution over goals given the observed actions.

In our work, we took the core components of these models to identify what kinds of actions never appear to pursue an instrumental goal. In section 1.1 we briefly describe the logic of inverse planning. Section 1.2 then explains how we used this framework to derive our theory predictions. Finally, section 1.3 shows how we implemented our computational model to quantify communicativeness in our studies.

1.1 Inverse planning through Markov Decision Processes

More detailed explanations of inverse planning can be found in [1, 2, 4].

In simple two-dimensional worlds, agents' behavior can be modeled as a Markov Decision Process (MDP) [5]. MDPs consist of a set of states S, a set of actions A, a reward function R, and a transition function T. At any time point, the world is in a state $s \in S$ that encodes all the information in the environment including the agent's position and the location of different objects. At each time step, the agent can take an action $a \in A$, which changes the state of the world. The relationship between actions and states is given by the transition function T, where $\mathrm{T}(s, a, s')$ is the probability that the world will be in state s' when the agent takes action a in state s. Each combination of states and actions yields a reward, given by $\mathrm{R}(s)$[1], which can be positive or negative.

Using this framework, it is possible to derive the set of actions, called a policy, that maximizes the agent's long-term rewards [see 5, for a detailed introduction]. This policy $\pi_R : A \rightarrow S$ is given by

$$\pi_R(s) = \arg\max_{a \in A} \sum_{s' \in S} T(s, a, s') V_R^*(s'). \quad (1)$$

where V_R^* is the optimal value function, given by

$$V_R^*(s) = R(s) + \max_{a' \in A} \lambda \sum_{s' \in S} T(s, a, s') V_R^*(s'). \quad (2)$$

Here, $\lambda \in (0, 1)$ is a parameter that determines the agent's future discount. This parameter is necessary for the planning algorithm to converge,

[1] The reward function can also depend on the action. Because this does not make a difference in our analyses, we use the simpler formulation.

and it helps creates efficient plans (as shorter paths reach rewards faster). Given the ability to transform reward functions into action plans or policies (Eqs. 1–2), goals can be formalized as reward functions that have a positive reward in the target state, and negative rewards (i.e., a cost) in all remaining states.

Instrumental goal attribution can then be formalized as an inverse planning problem: inferring the reward function behind an observed policy trace (i.e., a sequence of actions executed from a policy). Given a set of observed actions, the probability that an agent had unobservable reward function R is given by

$$p(R|A) \propto p(A|R)p(R) \tag{3}$$

Under a strict expectation that agents' actions are maximally efficient, the likelihood function can be set to 1 whenever an action matches the one predicted by the optimal policy, and 0 otherwise. However, because agents can often be inefficient due to distraction or planning errors, inverse planning relaxes the expectation for optimal efficiency by defining the probabilistic policy

$$\pi_R(a|s) \propto \exp(\beta \sum_{s' \in S} V_R^*(s')T(s,a,s')) \tag{4}$$

where $\beta \in [0, \infty)$ is the parameter that determines the agent's rationality. When $\beta = 0$, Eq. 4 produces a uniform distribution over actions, regardless of state. As β increases, the probabilistic policy converges to the optimal one.

1.2 Rarity and repetition reveal that the movement is not world-directed

Using this framework, we can formalize our hypothesis—that communicative actions are shaped to reveal the absence of an instrumental goal—as equivalent to the problem of finding sequences of actions that have a low probability of being generated by any instrumental goal.

For simplicity, we focus our analysis on grid-worlds like the ones typically used in action understanding tasks, and where inverse planning has been extensively tested [1, 2, 4]. According to our hypothesis, a communicative set of actions $\vec{a}$ going through states $\vec{s}$ ought to minimize

$$p(\vec{a}, \vec{s}|R)p(R) \tag{5}$$

for any reward function R that encodes an instrumental goal. Because in Markov Decision Processes the policy depends only on the current state, then

$$p(\vec{a}, \vec{s}|R) = \prod_{i=1}^{N} \pi_R(a_i|s_i). \tag{6}$$

That is, the likelihood of a sequence of actions in a sequence of states is given by the product of the probability of each action in each state.

Suppose that agents' actions change the state of the world deterministically such that, for instance, an agent in a grid world will always move north when taking action MOVE NORTH. Under this assumption, we can rewrite the transition function as a function $T : S \times A \to S$ that maps combinations of states and actions onto the next state. Taking Eq. 6 and replacing $\pi_R(a_i|s_i)$ with its definition (Eq. 4) results in

$$p(\vec{a}, \vec{s}|R) \propto \prod_{i=1}^{N} \exp(\beta V_R^*(T(s_i, a_i))). \tag{7}$$

where N is the number of observed actions. If a communicative action aims to minimize Eq. 7, then it must minimize V_R^* (as all other values are constants), for any reward function R that encodes an instrumental goal.

In grid-worlds, an instrumental reward function with goal state s_r (i.e., one where the reward is negative for all state except target state s_r) creates a graded value function such that $V(s') > V(s)$ whenever the distance $D(s', s_r)$ is greater than $D(s, s_r)$. In other words, states that are physically closer to the target state have higher values. This means that the sequence of actions that minimizes V_R^* is one where each action is selected so as to maximize the distance between the agent and the rewarding state (i.e., select actions that maximize $D(s, s_r)$ where s is the resulting state from the action, and s_r is the state associated with the instrumental goal). Simply put, moving away from a goal is what most directly reveals that the agent is not pursuing that goal.

Suppose now that there is a family of reward functions $\mathbb{R}$, each encoding an instrumental goal (i.e., $R(s) < 0$ in all states but one, where $R(s) > 0$, for all $R \in \mathbb{R}$). A communicative movement $\vec{a}$ taken in states $\vec{s}$ should minimize

$$\max_{R \in \mathbb{R}} p(\vec{a}, \vec{s}|R) \tag{8}$$

That is, the reward with the highest likelihood should be as low as possible.

As noted above, for any instrumental goal, its likelihood can be minimized from moving away from the goal. Therefore, if the distribution of instrumental goals is such that a single action can distance the agent from all instrumental goals, then this action minimizes Eq. 8. We call this type of action a 'rare' action in that it is unlikely under any possible goal.

Rare actions are not possible only when every action has at least one instrumental goal that they move towards. In this case, a communicative movement must minimize Eq. 8 and, at some point, moves away from each possible goal. If an initial action moves the agent towards a goal, then this goal's posterior probability will increase. To counter that, the next action must distance the agent from that same goal and must therefore reverse

itself. Intuitively this corresponds to an agent moving in one direction to reveal that they are not pursuing the goals behind them, and then reversing their direction to reveal that they also not pursuing the goals that an observer might consider after seeing the first movements. We call this type of solution ‘repetitive.’

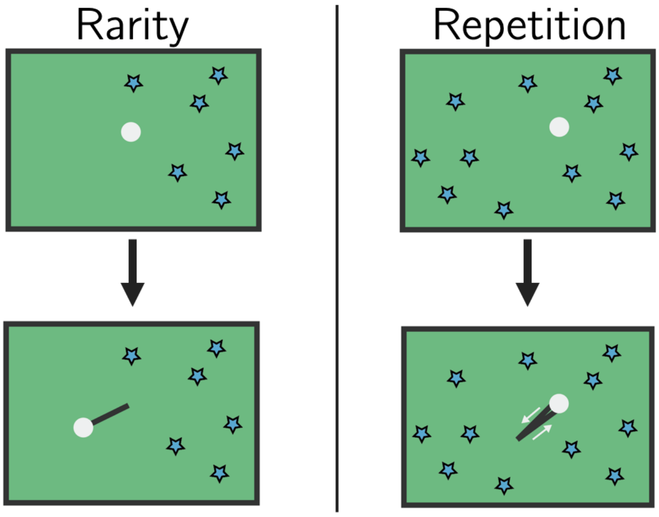

Figure 1: Visual schematic of intuitions behind rarity and repetitions as cues that an agent is not acting towards a physical goal.

1.3 Model implementation

To generate quantitative model predictions for Study 1, we implemented a simple model that computed the probability that an agent was acting towards an instrumental world-directed goal, given their actions, as given by

$$p(W|a) \propto p(a|W)p(W) \tag{9}$$

where

$$p(a|W) = \sum_{R \in R} p(a|W)p(R|W) \tag{10}$$

In Studies 1-4 we told participants that the agent was equally likely to be pursuing an instrumental or a communicative goal and we thus assumed that $p(W) = 0.5$. In addition, because participants could not see the potential goals or knew any information for their distribution, we assumed that every possible state could be a state with an instrumental goal (such that the number of reward functions equals the number of states) with a uniform prior distribution.

To obtain the model prediction for each video, we transferred the movements onto a grid-word of size 121×121, with the agent always beginning at the center of the map, in state $(60, 60)$. This results in 14641 possible goal states. We thus approximated equation 10 by only considering rewards at every tenth state, omitting the starting point, which resulted in 1464 possible instrumental goals. For each potential goal, we used a traveling cost of 1 (and a traveling cost of 1.414 for diagonal actions, following the pythagorean theorem), a reward of 10, a discount parameter of 0.95, and softmax parameter $\beta = 4$ (or, equivalently $\tau = 0.25$).

The paths from Study 1 were entered into the model by transforming each primitive path unit into three discrete actions. Straight paths were coded as three straight actions, and diagonal paths were coded as three diagonal actions. Curved paths were coded were approximated as a combination of two straight actions and a diagonal one. For instance, a curved path moving in the northeast direction was coded as 'move north', 'move northeast', 'move east.' Because each path in Study 1 consisted of four connected primitive paths, each path input to the model consisted of twelve actions.

1.3.1 Model predictions

Because each trajectory consisted of a series of actions, each with probability between 0 and 1, the likelihood term has an exponential decay. To cushion this effect we represent these probabilities in log-space.

Our model predictions and participant judgments are in different scales. To make the analyses easier to interpret, we first applied a linear transformation by training a linear regression to predict participant judgments based on model predictions, the resulting transformation was

$$\text{Participant judgment} = -2.2283 - 0.2703 \log(p(a|W)) \tag{11}$$

Supplemental Information for Studies 1-3 and Explanation Control

Stimuli for all studies available at https://osf.io/ehb48/.

Procedure Details

After completing the Study 1 replication, Studies 2-3, the Explanation Control, and their replications, participants were asked to confirm that they watched all the videos and were explicitly told that their answer would not affect their compensation. Answering no would have resulted in exclusion, however, all participants reported watching all of the videos. Participants were also asked to describe any strategies that they used while providing their responses (see https://osf.io/ehb48/ for responses to post-test questions).

Study 1

Cover Story. All participants read the following cover story: *There is an anthropologist doing research on a remote island. Once a week, a helicopter flies over the island and the anthropologist has to signal the helicopter if he needs additional supplies. Because the tree cover on the island is so thick, the helicopter operator can only track the anthropologist's movements using an infrared camera. The camera is very good at capturing motion. Because of this, the anthropologist signals different requests using previously agreed-upon walking movements. Some days, the anthropologist needs supplies and will move to communicate a message to the helicopter. Other days, the anthropologist will not need to communicate anything and will continue doing his research and maintaining his base camp. You will be shown videos of the anthropologist's movements on different days and asked to rate how likely you think it is that the anthropologist was communicating something to the helicopter that day on a scale of one (definitely not communicating) to seven (definitely communicating).*

Data Analysis. We evaluated the strength of the relationship between each path's rarity and its average communicativeness rating with a Pearson's correlation and a 95% bootstrapped confidence interval ($r = 0.80$, $CI_{95\%} = .67, 1$; see Supplementary Fig. 2). Then, a mixed effects model was used to predict participant judgements as a function of the path's rarity with random intercepts for path class (see main text methods for class names). Participant was not included as a random intercept to avoid having a singular fit for the model. Participants' judgments were significantly predicted by the rarity of the path, such that more rare paths were seen as more communicative ($\beta_{rarity} = 1.90$, $p < 0.001$).

Study 1 Replication. Thirty MTurk participants were recruited for a pre-registered replication of Study 1 (AsPredicted #12593). Stimuli, experimental protocols, data analyses, and statistical tests were identical to those detailed above. Participants judged more rare paths as more communicative (Pearson's correlation: $r = 0.87$, $CI_{95\%} = .79, 1$; mixed effects model: $\beta_{rarity} = 3.16$, $p < .001$; see Supplementary Fig. 2).

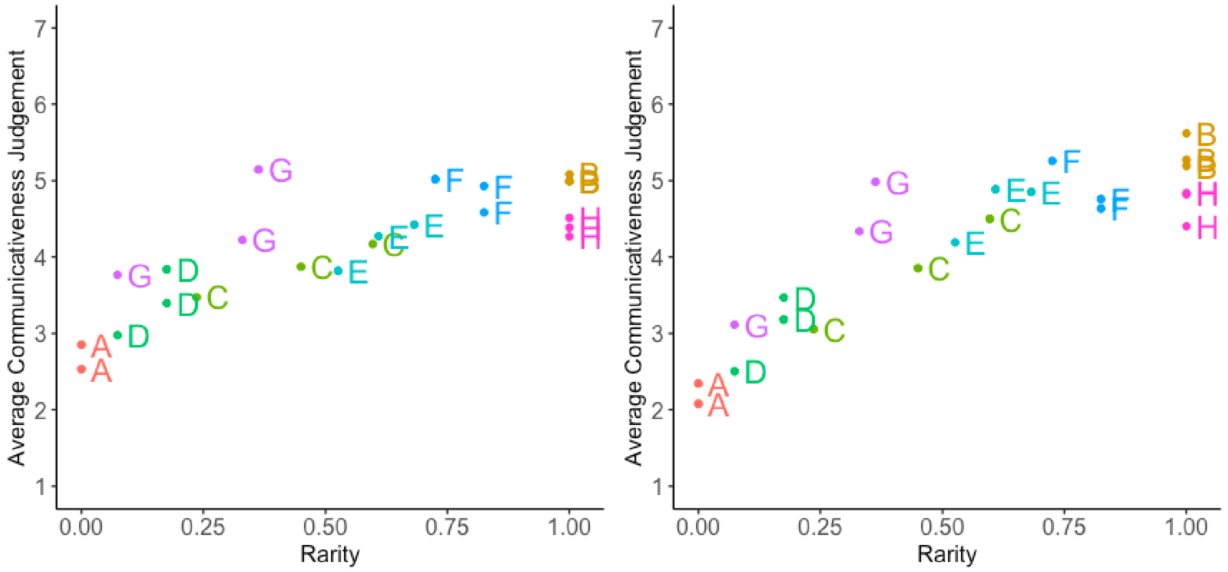

Supplementary Fig 2. Results from Study 1(left) and the Study 1 Replication (right). Average communicativeness judgments (y-axis) compared to the path's rarity (x-axis). Letters represent the path's movement class.

Testing for Effects of Distance as a Potential Confound. Study 1 began by operationalizing our notion of rarity in terms of inefficiency (although our computational model uses a richer definition in terms of non-world-directedness), defined as the ratio between the length of the observed path, and the length of the shortest possible path to reach the end point. Therefore, rarity in this study correlates with path length. This raises the possibility that participant communicative judgments were driven by overall distance travelled. To test for this possibility, we computed the distance travelled for each path and correlated it against participant communicativeness judgments (combining Study 1 and its replication). The correlation between communicativeness and distance traveled was $r = 0.56$ (Supplementary Fig. 3), which is substantially lower than the correlation we observed between rarity and communicativeness ($r = 0.85$).

Additionally, we ran linear mixed effects models for Study 1 and its replication examining the relationship between participant judgements, distance, and rarity. The model predicted participant judgments from path distance and rarity with random intercepts for path class and participant. In this regression, distance marginally predicted participant judgements ($\beta_{distance} = 0.004$, $p = 0.06$), but the beta coefficient was orders of magnitude less than the beta coefficient of rarity ($\beta_{rarity} = 2.40$, $p < 0.001$). Thus, even when partialling out the effect of distance on communicativeness judgements, rarity remains a strong predictor of communicativeness judgements. The small effect of distance may hint at a possible separate effect on

communicativeness (perhaps as a proxy for effort), but more systematic manipulations focusing on distance are needed to support this hypothesis.

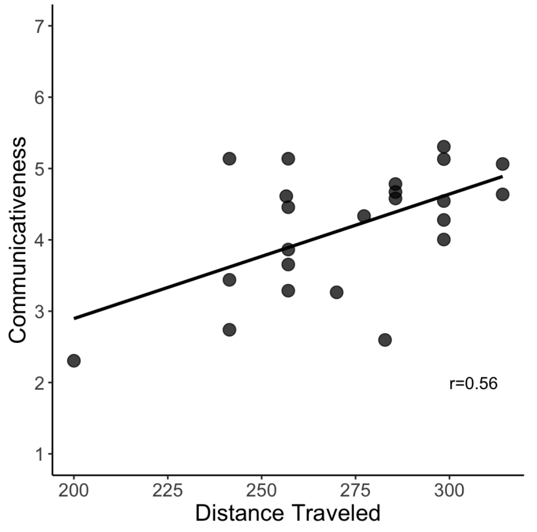

Supplementary Fig 3. Average communicativeness judgements (y-axis) as a function of the distance traveled by the path (x-axis) in Study 1 combined with its replication.

Disentangling the role of rarity and path length in Study 1 is difficult as these two naturally correlate. Note, however, that Studies 2 and 3 further rule out the possibility that participants responded based on path length. In Study 2, we created three versions of each path, which varied in the number of repetitions while keeping the total distance traveled constant. Here, participants rated movements with more repetitions are more likely to be communicative, despite being matched in distance to the movement with fewer repetitions. In Study 3, the distance traveled by each paired path in the bordered and unbordered conditions was held constant, but participants rated the unbordered condition paths as significantly more likely to be communicative than their bordered counterparts. Thus, Studies 2 and 3 provide even stronger evidence for rarity rather than distance as the driving factor behind communicativeness judgements. These results show that distance alone cannot explain participant communicativeness judgments.

Study 2

Data Analysis. A mixed effects model was used to predict participants' communicativeness judgments based on the rarity of the basic version of the path and the path's condition (zero repetitions, one repetition, two repetitions, coded numerically) with random intercepts included for each participant. Crucially, Study 2 replicated the findings of Study 1, such that rarity of the

unrepeated version of each path also significantly predicted communicativeness judgements (β_{rarity} = 1.16, $p < .001$).

Study 2 Replication. Thirty MTurk participants each were recruited for pre-registered replication of Study 2 (AsPredicted #13923). In addition to replicating the significant main effect of number of repetitions, the Study 2 replication also found that the rarity of the unrepeated version of the paths significantly predicted participant judgements as well (β_{rarity} = 1.09, $p < .001$).

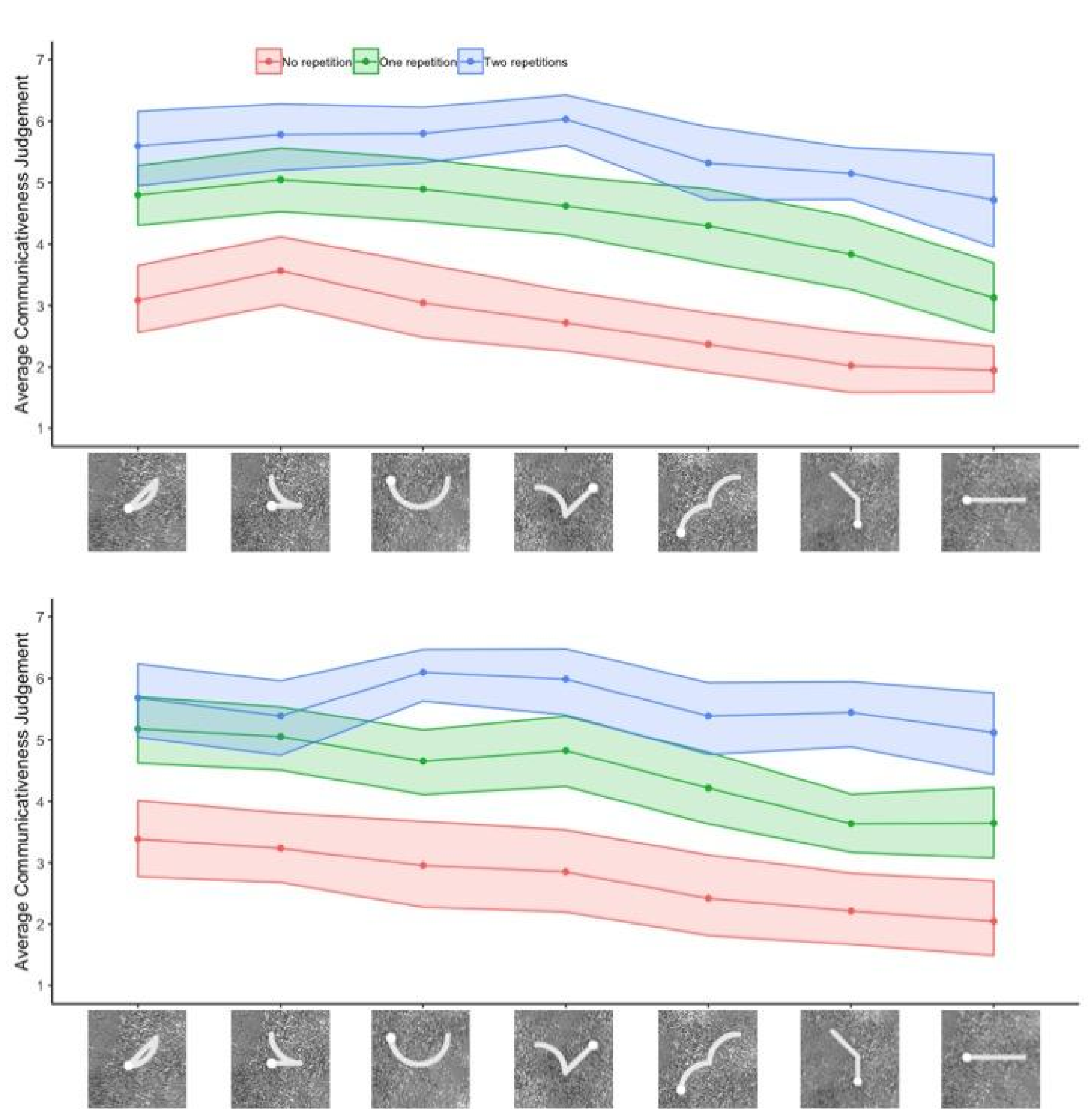

Supplementary Fig 4. Results from Study 2 (top) and the Study 2 Replication (bottom). Average

communicativeness judgments (y-axis) of the unrepeated, one repetition, and two repetition versions of each path. Paths are ordered from left to right in order of decreasing rarity of the unrepeated version of the path. Error bands represent 95% bootstrapped confidence intervals.

Study 3

Data Analysis. Using a mixed effects model, we predicted participants' communicativeness judgments as a function of the path's rarity, path condition (bordered vs. unbordered), and their interaction. Participant and path class were included as random intercepts. Communicativeness ratings were significantly predicted by whether the path was bordered or unbordered ($\beta_{condition}$= 0.87, p = .002), path rarity (β_{rarity} = 1.30, p = .008), and the interaction between condition and path rarity ($\beta_{condition:rarity}$= 1.14, p = .002).

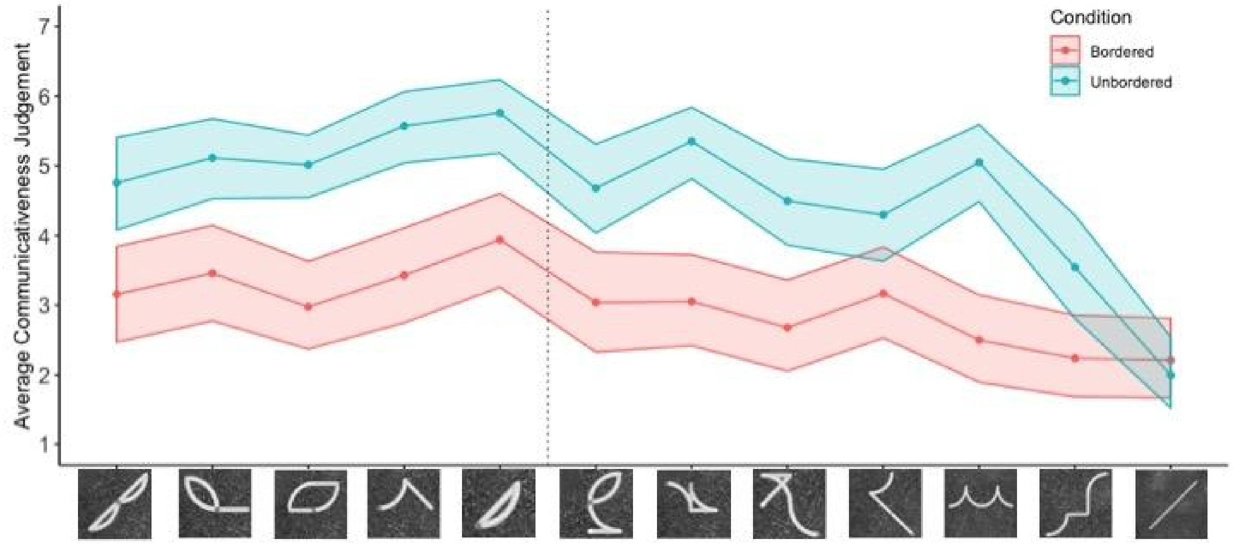

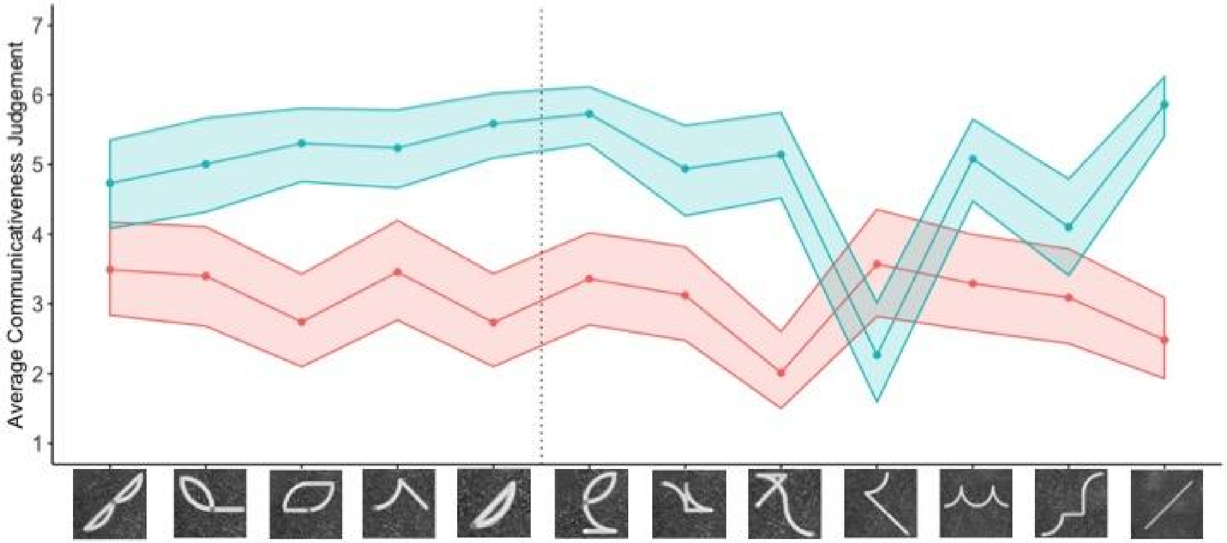

Supplementary Fig 5. Results from Study 3 (top) and the Study 3 Replication (bottom). Average communicativeness judgments (y-axis) of the border and unbordered versions of each path. Paths to the right of the dotted line are ordered from left to right in order of decreasing rarity. Paths to the left of the dotted line are maximally rare because they all start and end in the same location. Error bands represent 95% bootstrapped confidence intervals.

Study 3 Replication. Thirty MTurk participants were recruited for the pre-registered replication of Study 3 (AsPredicted #13925). In the Study 3 replication, communicativeness ratings were significantly predicted by whether the path was bordered or unbordered ($\beta_{condition}$= 1.23, $p < .001$) and the interaction between condition and path rarity ($\beta_{condition:rarity}$= 0.92, $p = .015$). However, in this replication, path rarity alone did not significantly predict communicativeness judgments (β_{rarity} = 1.02, $p = .110$).

Explanation Control

Cover Story. This following revised cover story was used for the Explanation Control: *There is an anthropologist doing research on a remote island. Once a week, a helicopter flies over the island and records the anthropologist's movements. Because the tree cover on the island is so thick, the helicopter operator can only track the anthropologist's movements using an infrared camera. The camera is very good at capturing motion, but cannot see any objects on the island. You will be shown videos of the anthropologist's movements on different days and asked to guess what he was doing.*

Responses. Participants' descriptions of what the anthropologist was doing are available on OSF (https://osf.io/ehb48/). To ensure that participants understood and were fully engaging with the task, two blind coders and a tiebreaker coded whether participants' written responses were consistent with the scenario presented in the cover story. Of the 1,308 responses collected in the Explanation Control and the Explanation Control replication, only 14 were coded as unreasonable or nonsensical (for example, one participant said that the anthropologist was flying

in an airplane, which is inconsistent with the scenario presented). This coding procedure was not used as an exclusion criteria; it was only used as a validation to ensure that the results could not be explained due to task misunderstanding.

Data Analysis and Statistics. The difficulty ratings across participants were averaged for each path, generating one average difficulty score for each of the 23 videos. Then, the linear relationship between each path's average difficulty of explanation score and path rarity (calculated in Study 1) was examined using a Pearson's correlation and a 95% bootstrapped confidence interval. Rarer paths did not receive correspondingly higher difficulty of explanation scores ($r = 0.10$, $CI_{95\%} = -.31, 0.56$). A reviewer requested p-values for this effect and thus we also include a mixed effects model predicting participant judgements as a function of the path's rarity with random intercepts for path class and participant ($\beta_{rarity} = 0.21$, $p = 0.427$).

Replication. Thirty MTurk participants were recruited for the pre-registered replication of Explanation Control (AsPredicted #13961). In the replication of Explanation Control, we found a stronger correlation between the difficulty of explanation ratings and path rarity ($r = 0.38$, $CI_{95\%} = 0.14, 0.83$) than in the original study. Notably, though even if this correlation was statistically significant, the strength of the correlation is not as strong as the correlation between communicativeness (Study 1) and rarity in Study 1 ($r = 0.85$). Additionally, difficulty of explanation was not significantly predicted by the path's rarity in a mixed effects model with random intercepts for path class and participant ($\beta_{rarity} = 0.40$, $p = 0.188$). Therefore, difficulty inferring non-communicative goals for movements cannot explain all of the variability associated with judgements of communicativeness.

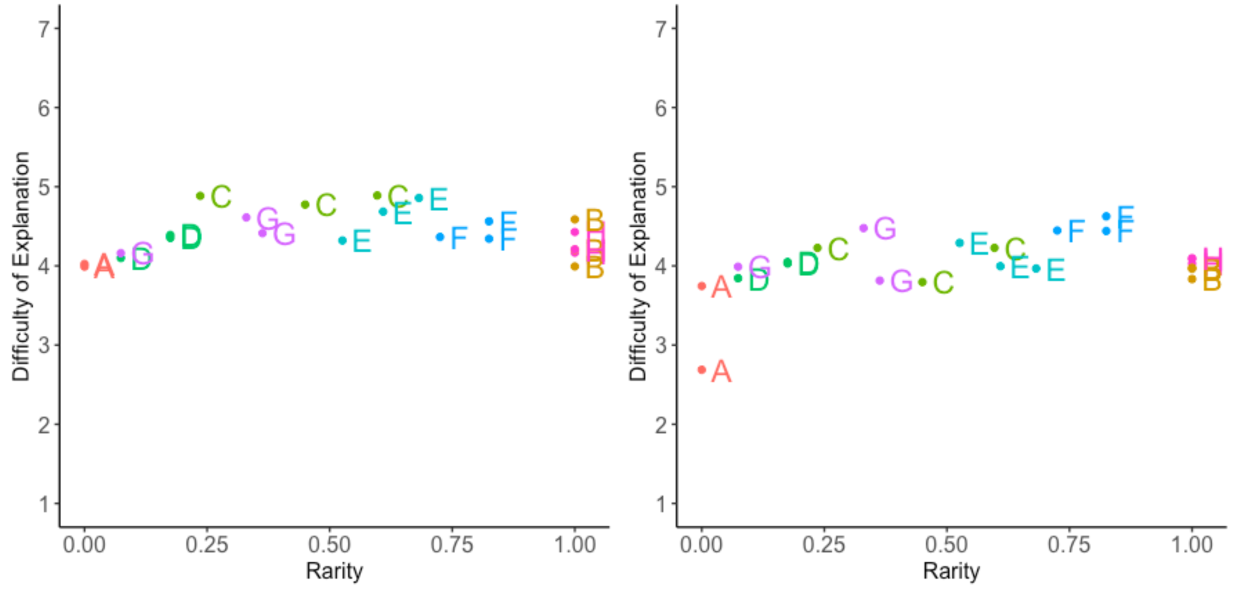

Supplementary Fig 6. Results from Explanation Control (left) and the Explanation Control Replication (right). Average difficulty of explanation judgments (y-axis) compared to the path's rarity (x-axis). Letters represent the path's class.

As a check to see if participants understood the task, we also examined the variability in participants' ratings. If participants did not understand the test question, their ratings should be clustered around the midpoint (4 in our seven-point scale) with little variability (suggesting that participants did not know how to respond), or with the same variability across all trials. However, if participants understood the question, their responses should reflect the difficulty that they experienced when generating different non-communicative explanations. Although the average difficulty ratings for the Explanation Control clustered around 4, there was notable variation in participants' responses. As Supplementary Fig. 7 shows, participants did not uniformly favor entering the midpoint (4) for all trials, and all paths did not have the same distribution of answers. Instead, each trial shows a different pattern of responses. For instance, in trial *ihep* (see Supplementary Fig. 8 for explanation for trial names), participants showed a bimodal distribution, where roughly half of the participants found it easy to generate an explanation and half of the participants found it difficult. By contrast, in trial *abkj*, participants were equally likely to find the process of generating an explanation easy, average, or difficult. Altogether, this figure suggests that participant answers reflected how difficult they found it to generate explanations, rather than a poor understanding of the task.

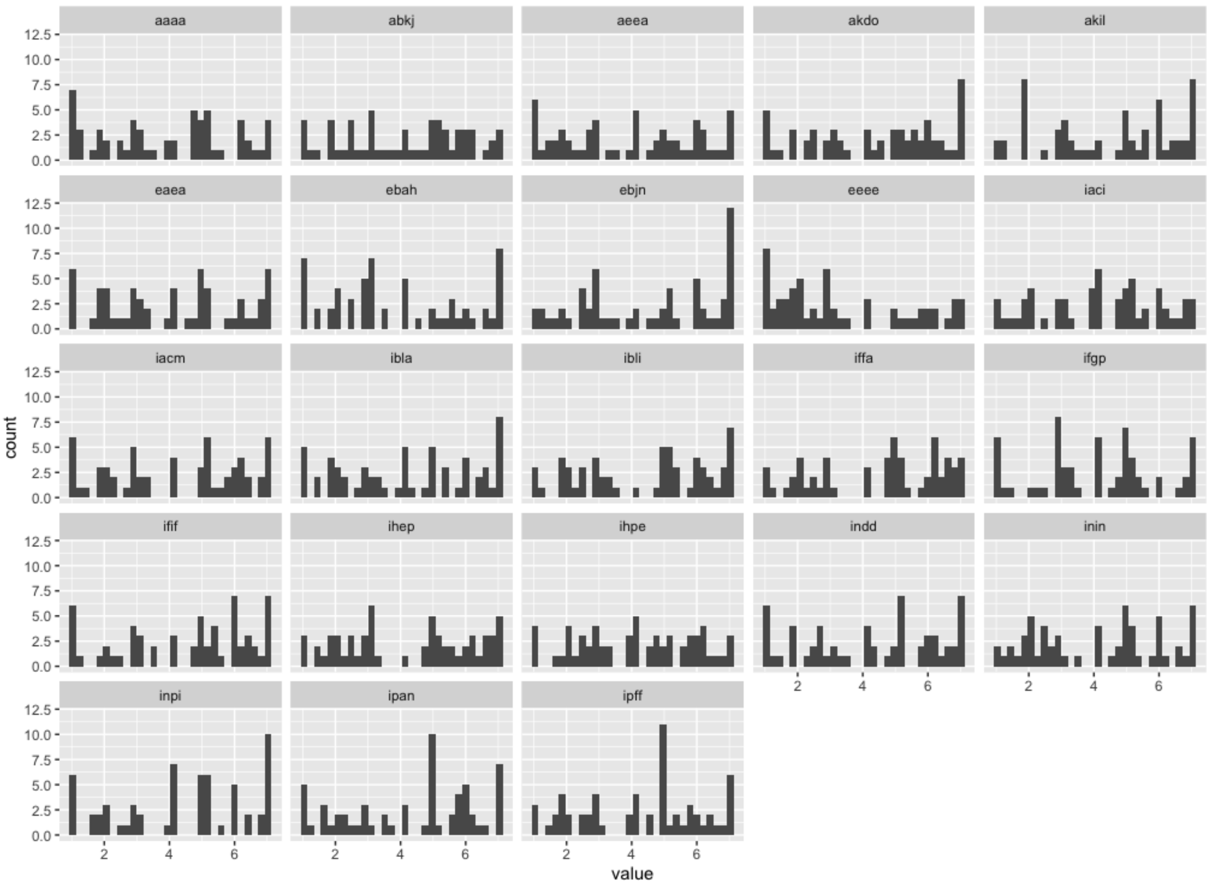

Supplementary Fig 7. Histograms of the participant ratings for each of the 23 stimuli showing the frequency (y-axis) of different difficulty ratings (x-axis). Each subplot label indicates the stimulus pathi video (see Supplementary Fig. 8 for an explanation of each label's meaning).

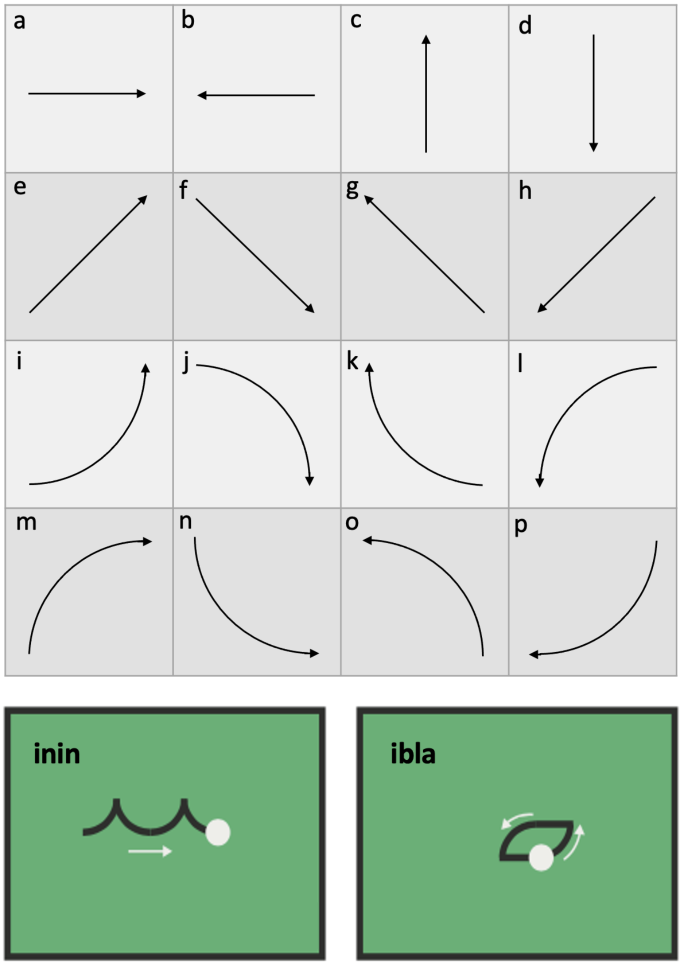

Supplementary Fig 8. An explanation of the naming convention in figure above. Each path in Studies 1-3 was made up of four primitiative paths from a set of 16 possible primitives shown in the grid above (See Methods Study 1-3 section "Stimuli" for a detailed explanation of path creation). Each primitive was assigned a letter; thus each path can be represented as a sequence of four letters. *inin* and *ibla* are two examples of paths and their corresponding letter sequences.

Supplementary Model Analysis

Data Analysis and Statistics. In order to test whether removing the six data points in classes B and D significantly improves the fit model beyond what one would expect from removing any six points, we conducted a permutation test with 10,000 samples. For each sample, we computed one correlation between the model predictions and the participant responses based on randomly removing six data points. This was repeated 10,000 times to test whether the improved correlation between the model and participant data resulting from removing points in classes B

and D was significantly different from the distribution of the correlations based on random removal of datapoints. We found that correlation resulting from the removal of data points in classes B and D was significantly different from the distribution of correlations based on random removal ($r = 0.90$, $CI_{95\%} = .84\text{-}1$, $p < 0.001$; Supplementary Fig. 9).

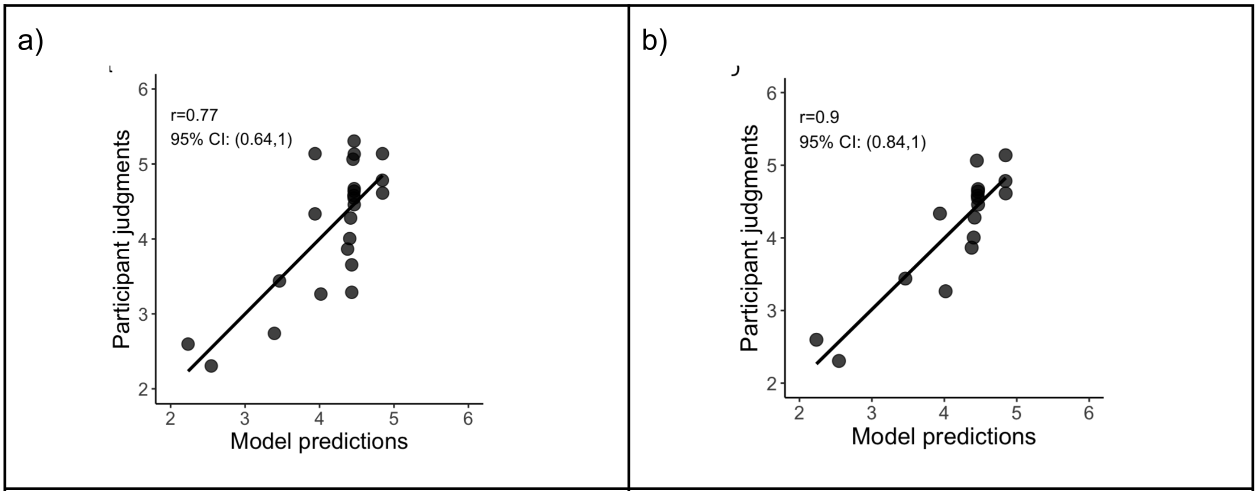

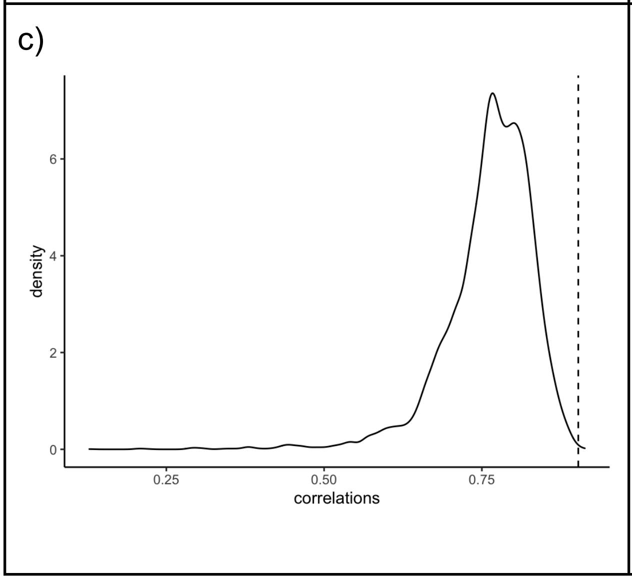

Supplementary Fig 9. a) a reproduction of Fig. 3b from the main text, which shows final model predictions (x-axis) against participant judgments (y-axis) from Study 1. Each point represents a path's model prediction (x-axis; negative log of probability of a world-directed goal given the full trajectory. b) b) Final model predictions (x-axis) against participant judgments (y-axis) from Study 1, excluding data from paths in categories B and D. c) A density plot of the results of our permutation test. The correlation coefficients resulting from randomly removing six data points (x-axis) against the frequency of those values in the 10,000 samples (y-axis). The dashed vertical line represents the correlation coefficient based on removing classes B and D ($r = 0.90$).

Supplemental Information for Studies 4-7

Stimuli for all studies available at https://osf.io/wxdka/.

Study 4 (AsPredicted #12589 and #12590)

Sample Size Rationale. Our sample size of 60 participants (30 for each video set) was decided on in order to have 30 judgements for each video since all participants viewed and rated all videos.

Cover Story. Participants read the following cover story: *You are going to see videos of Lucy. Lucy is from an island that is very remote. On Lucy's island, people also move their bodies to tell each other things, but on Lucy's Island they don't shake their head or give a thumbs up; they have a whole unique set of gestures. You will be shown videos of Lucy as she is mixing food. Sometimes Lucy stops mixing in order to use her hands to communicate something to her friend. Other times Lucy stops mixing, but is not communicating with her friend. You will be shown videos of Lucy's movements and asked to rate how likely you think it is that Lucy was communicating something to her friend with her movements on a scale of one (definitely not communicating) to seven (definitely communicating).*

Procedure Details. After completing the study, participants were asked to confirm that they watched all the videos and were explicitly told that their answer would not affect their compensation. Answering no would have resulted in exclusion, however, all participants reported watching all of the videos. Participants were also asked to describe any strategies that they used while providing their responses (see https://osf.io/wxdka/ for responses to post-test questions).

Data Analysis. The features of each movement were coded as follows: whether the movement was repetitive (yes or no) and whether the hand touched another part of the body (yes or no). This allowed us to examine the effects of these features and their interaction. First, the data from Study 4a with the low punctuality video set was analysed using a mixed effects model predicting participants' communicativeness judgments based on the movement's repetitiveness, physical contact, and their interaction; random intercepts were included for each participant and basic movement type. This full model was compared against a simpler model that did not include the interaction. The inclusion of the interaction did not significantly improve the fit ($p = 0.986$), therefore it was not included in the final model.

The data from Study 4b with the natural punctuality video set was also analyzed with a mixed effects model predicting participants' communicativeness judgments based on the movement's repetitiveness, physical contact, and their interaction; random intercepts were included for each participant and basic movement type. In this model, the interaction between rarity and repetition

was trending towards significance ($\beta_{rarity:repetition}$ = -0.43, p = .069). Additionally, data from the two studies were combined in order to analyze whether communicativeness judgements were predicted by the different demonstrators. For this analysis, we ran a mixed effects model predicting participants' communicativeness judgments based on the demonstrator (low punctuality vs. natural punctuality) with random intercepts included for participant and basic movement type.

Weirdness Control

Sample Size Rationale. Our sample size of 30 participants was decided on in order to have 30 judgements for each video since all participants viewed and rated all videos.

Stimuli. The low punctuality videos used in Study 4a were used in this study.

Cover Story. Participants recruited from MTurk read the following cover story: *You are going to see videos of Lucy. Lucy is from an island that is very remote. On Lucy's island, people also move their bodies to tell each other things, but on Lucy's Island they don't shake their head or give a thumbs up; they have a whole unique set of gestures. You will be shown videos of Lucy as she is mixing food. Sometimes Lucy stops mixing in order to use her hands to communicate something to her friend. Other times Lucy stops mixing, but is not communicating with her friend. You will be shown videos of Lucy's movements and asked to rate how weird her movements are on a scale of one (Not weird) to seven (Extremely weird).*

Procedure Details. After completing the study, Amazon Mechanical Turk participants were also asked the same post-test questions described in Study 4.

Data Analysis. Movement features were coded as described in Study 4. First, the data from this study was analysed using a mixed effects model predicting participants' weirdness judgments based on the movement's repetitiveness, and physical contact; random intercepts were included for each participant and basic movement type. Participants gave significantly higher judgements for movements that were repetitive ($\beta_{repetition}$: 0.43, p < .001), but not for movements that were rare (β_{rarity}: 0.07, p = .444).

Then, the data from Study 4a was combined with this data from the Weirdness Control to analyze whether the question type (communicativeness or weirdness) significantly predicted the effects of rarity and repetition. The combined data was analysed using a mixed effects model predicting participants' judgments based on the question (communicativeness or weirdness), the movement's repetitiveness, the movement's physical contact, the interaction of question and repetitiveness, and the interaction of question and rarity; random intercepts were included for each participant and basic movement type. According to the model, participants were marginally more likely to give a lower judgment when the question was about communicativeness ($\beta_{question}$: -0.50, p = .062) and, as reported above, participants gave significantly higher judgements for

movements that were repetitive ($\beta_{repetition}$: 0.43, $p < .001$), but not for movements that were rare (β_{rarity}: 0.07, $p = .497$). Most importantly, the size of the effect was significantly predicted by the interaction of question with rarity ($\beta_{question:rarity}$: 1.48, $p < .001$) and repetition ($\beta_{question:repetition}$: 0.39, $p = .007$).

Familiarity Control (AsPredicted #12589 and #12590)

Sample Size Rationale. Our sample size of 60 participants (30 for each video set) was decided on in order to have 30 judgements for each video since all participants viewed and rated all videos.

Procedure Details. After completing the study, Amazon Mechanical Turk participants were also asked the same post-test questions described in Study 4.

Cover Story. Participants recruited from MTurk read the following cover story: *You will be shown videos of a woman as she is mixing food. In each video, she will stop mixing in order to use her hands to communicate something to her friend. You will watch the videos and be asked if you recognize the gesture and what you think that the gesture means.*

Responses. Although participants' descriptions of what they thought the gesture meant were not analysed, they are available on OSF (https://osf.io/wxdka/).

Data Analysis. Because responses were not significantly predicted by demonstrator type in Study 4, the communicativeness responses and familiarity ratings from the two demonstrator sets were combined for these analyses. First, the data from this study was analysed using a mixed effects model predicting participants' familiarity judgments based on the movement's repetitiveness, and physical contact; random intercepts were included for each participant and basic movement type. Neither rarity (β_{rarity}: 0.04, $p = .598$) nor repetition ($\beta_{repetition}$: -0.0002, $p = .998$) significantly predicted participant judgements.

We also conducted a similar analysis to the one used in the Weirdness Control. The data from Study 4a and 4b (since this Familiarity Control used both video sets) was combined with this data from the Familiarity Control to analyze whether the question type (communicativeness or familiarity) significantly predicted the effects of rarity and repetition. The combined data was analysed using a mixed effects model predicting participants' judgments based on the question (communicativeness or familiarity), the movement's repetitiveness, the movement's physical contact, the interaction of question and repetitiveness, and the interaction of question and rarity; random intercepts were included for each participant and basic movement type. According to the model, question ($\beta_{question}$: 0.31, $p = .153$), rarity (β_{rarity}: 0.04, $p = .631$), and repetition ($\beta_{repetition}$: -0.0002, $p = .998$) were not significant predictors. Most importantly, the size of the effect was

significantly predicted by the interaction of question with rarity ($\beta_{question:rarity}$: 1.55, $p < .001$) and repetition ($\beta_{question:repetition}$: 1.05, $p < .001$).

Study 5 (AsPredicted #13223, #13317, #17032, #13388)

Sample Size Rationale. Our sample size of 120 Tsimane' participants (for the initial study using the low punctuality video set) was decided on in order to have 30 participants per trial order. We reduced the sample size to 60 for the follow-up in which Tsimane' participants rated the videos from the natural punctuality video set. This was based on a power analysis of the results from the sample tested on the low punctuality video set.

Exclusions. Forty Yale undergraduates (AsPredicted #17032), forty San Borjans (#13317), and 180 Tsimane' (#13223, #13388) participated in this study. An additional 27 Tsimane' participants failed to correctly answer the warm-up questions and were excluded from further participation in the study. We also excluded all data from participants from a specific Tsimane' community (22 participants) because during testing we had reason to believe that participants had discussed the study, which caused later participants to point to videos before being asked questions.

Of the cohort of Tsimane' that were run on the experimental procedure described above using the videos from the natural punctuality video set (AsPredicted #13388), nine additional participants were excluded for failing the warm-up questions.

Script. "You are going to watch two videos. In one of the two videos the person is saying hello. You have to indicate in which video the person is saying hello" [Participant watched the first pair of warm-up videos].

[If participant answered incorrectly] "How would you say hello with your movements?" [The videos were played again]. "In which video is the person saying hello?" [If the participant answered incorrectly a second time they were excluded from further participation in the study. If they answered correctly, then they moved on to the second pair of warm-up videos.]

"In one of the two videos the person is saying no. You have to indicate in which video the person is saying no" [Participant watched the second pair of warm-up videos].

[If participant answered incorrectly] "How would you say no with your movements?" [The videos were played again]. "In which video is the person saying no?" [If the participant answered

incorrectly a second time they were excluded from further participation in the study. If they answered correctly, then they moved on to the test trials.]

The order of the warm-up videos was counterbalanced across participants.

"Now you are going to watch videos from another part of the world that you are not familiar with. You have to indicate in which video the person is communicating with their movements."

Data Analysis. We conducted two monte carlo permutation tests with 10,000 samples for each population. For each participant, we computed their score for the task based on the number of times that they responded that the demonstrator was trying to communicate in repetitive or rare videos (this constitutes a correct choice) divided by 3, the total number of trials for each type of video comparison. We then averaged together participants' scores for the rare videos and separately averaged their scores for the repetitive videos. Two permutation tests were then conducted (one for rarity and one for repetition) by shuffling each participant's responses, computing the scores of the shuffled participants, and calculating the average shuffled performance value 10,000 times to test whether the actual performance value was significantly different from the distribution of the average shuffled performance values. 95% bootstrapped confidence intervals were then calculated. Separate permutation tests were conducted on the data from the Tsimane' who viewed the natural punctuality video set.

Study 6 (AsPredicted #13960)

Sample Size Rationale. Initially, we aimed to complete sample sizes of 60 participants in order to have 30 judgements per video since each participant responded to half of the videos. However, due to time constraints, we only were able to collect 59 participants.

Script. "You are going to watch videos of someone cooking. Sometimes the person is cooking alone in the room. Other times, the person is cooking and there is someone else watching. For each video you have to watch what the person cooking does and say whether you believe that the person is alone or whether there is someone else in the room watching. [After watching the first warm-up video] In this video, the person was cooking and then stopped to kill a mosquito. Therefore, it is possible that the person was alone in the room. [After watching the second warm-up video] In this video, the person was cooking and then stopped to point at something. Therefore, it is possible that there was someone else in the room watching. In half of the videos the person is alone and in half there is another person there. You will not be able to see the other person when they are there. You have to guess based on what the person cooking does."

The order of the warm-up videos was counterbalanced across participants.

Procedure Details. After completing the study, Amazon Mechanical Turk participants were also asked the same post-test questions described in Study 4.

Data Analysis. Because we wanted to ensure that participants understood the task directions, we excluded data from participants (26 Tsimane' participants, 5 US participants) that either responded that the demonstrator was alone in all the videos or that there was someone else in the room for all the videos. This pre-registered exclusion criteria was also intended to exclude participants who were not engaging with the task. Using the data from the remaining participants, we conducted two monte carlo permutation tests with 10,000 samples for each population. For each participant, we computed one score on the task based on the number of times that they responded that the demonstrator was alone in the room for the basic videos (this constitutes a correct choice) divided by 3, the number of basic video trials. We also computed a second score based on the number of times that they responded that there was someone else in the room for the rare + repetitive videos (this constitutes a correct choice) divided by 3. We then averaged together participants' scores for the basic videos and separately averaged their scores for the rare + repetitive videos. Two permutation tests were then conducted (one for basic and one for rare + repetitive) by shuffling each participant's responses, computing the scores of the shuffled participants, and calculating the average shuffled performance value 10,000 times to test whether the actual performance value was significantly different from the distribution of the average shuffled performance values. 95% bootstrapped confidence intervals were then calculated.

Study 7 (AsPredicted #26450)

Sample Size Rationale. We collected data from as many Tsimane' participants as possible. Therefore, due to time constraints, our final Tsimane' sample is comprised of 32 participants. Our piloting of the study with US participants revealed a tendency to describe the motion in terms of its physical characteristics rather than the goals motivating the movement. Since we planned to exclude these low-level descriptions, we increased our sample size to 100. Because each participant responded to half of the videos, this allowed us to get 50 judgements per video of which we excluded explanations that were not descriptions of communicative or world-directed goals.

Stimuli. The basic and repetitive + rare videos from the low punctuality video set from Study 4a were used. Videos were presented one at a time to participants. Tsimane' participants completed the experiment in person with the stimuli presented on an iPad. MTurk participants completed the study on their personal computers.

Script. "You are going to watch videos of someone cooking. The person is going to stop cooking and do something else. [After watching the first warm-up video] For example, in this video, the person is cooking and kills a mosquito. [After watching the second warm-up video] For example, in this video, the person is cooking and points to something. For each video you have to say what the person was doing when the person stopped cooking."

The order of the warm-up videos was counterbalanced across participants.

Procedure Details. After completing the study, Amazon Mechanical Turk participants were also asked the same post-test questions described in Study 4.

Responses. Participants told their responses in Tsimane' to an interpreter who reported the responses in Spanish. The experimenter then translated the responses into English for coding.

Coding Scheme. The responses from participants were categorized by two coders blind to the stimuli information. Coders categorized each response into one of four categories: *descriptive*: the response only references the low-level physical movements of the action (e.g., "The person puts their left hand under their right hand and makes a motion with their right hand"), *communicative*: the response describes a goal in which the demonstrator is trying to communicate with another person through her motions, *world-directed*: the response indicates that the demonstrator is trying to accomplish some physical, external goal (e.g., wiping something off of her face), and *other*: the response is nonsensical or does not fit into any of the categories listed above. Any category disagreements were settled by a third blind coder who selected between the two categories chosen by the first two coders. Because we are only interested in explanations that posited a goal for the demonstrator's movements, we only included communicative and world-directed responses in the analyses (see Supplementary Fig. 10 for distribution of all four response categories).

Data Analysis. In the text, we report the percent of communicative responses given by both populations for basic and rare + repetitive videos out of the total number of responses that were coded as either "communicative" or "world-directed" supplemented with 95% bootstrapped confidence intervals. We also calculated the percent of each response type given by each population for basic and rare + repetitive videos out of all responses (not just those that were coded as either "communicative" or "world-directed") and supplemented with 95% bootstrapped confidence intervals, which are presented in the tables below.

(A) Percent of Responses from Tsimane' Participants

Response Category	Basic Movements	Rare + Repetitive Movements
Communicative	14.58% of trials, $CI_{95\%}$=8.33–21.88%	57.29% of trials, $CI_{95\%}$=46.88–66.67%
World-directed	78.13% of trials, $CI_{95\%}$=69.79–86.46%	35.42% of trials, $CI_{95\%}$=26.04–44.79%
Descriptive	7.29% of trials, $CI_{95\%}$=2.08–12.50%	6.25% of trials, $CI_{95\%}$=2.08–11.46%
Other	0.00% of trials	1.04% of trials, $CI_{95\%}$=0.00–3.13%

(B) Percent of Responses from US Participants

Response Category	Basic Movements	Rare + Repetitive Movements
Communicative	4.67% of trials, $CI_{95\%}$=2.33–7.00%	49.00% of trials, $CI_{95\%}$=43.33–54.67%
World-directed	60.67% of trials, $CI_{95\%}$=55.33–66.00%	11.67% of trials, $CI_{95\%}$=8.33–15.33%
Descriptive	32.00% of trials, $CI_{95\%}$=27.00–37.33%	33.33% of trials, $CI_{95\%}$=28.33–38.67%
Other	2.66% of trials, $CI_{95\%}$=1.00–4.67%	6.00% of trials, $CI_{95\%}$=3.33–8.67%

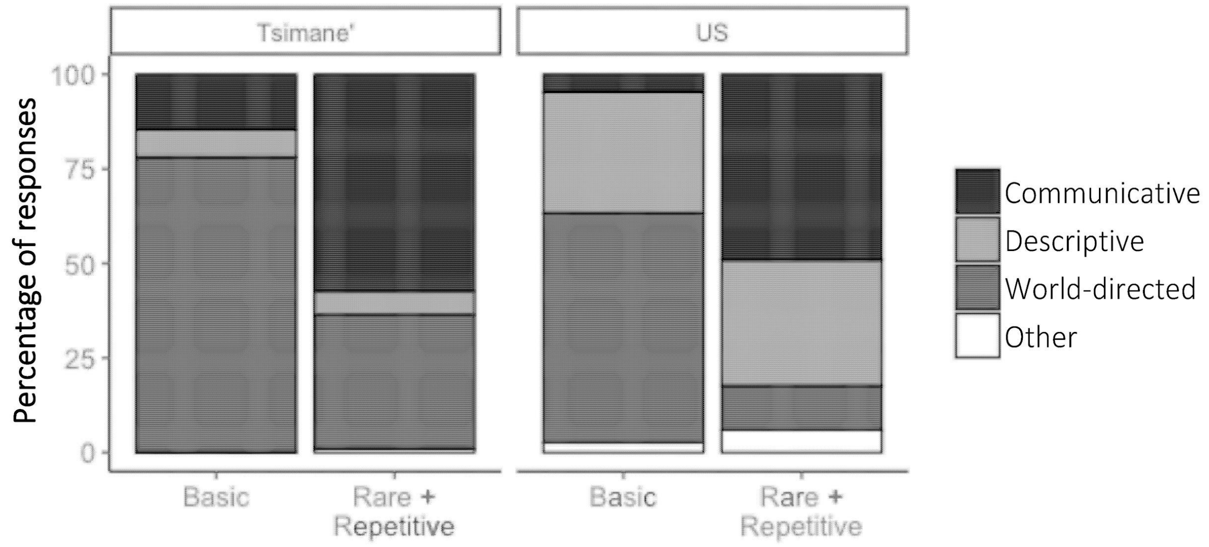

Supplementary Fig 10. Results from Study 7, including responses categorized as “descriptive” and “other”, which were excluded from our main analyses.

Tsimane’ Demographic Analysis

A priori, we did not expect that age, gender, or schooling should affect people’s judgements about novel communicative actions. However, in response to a reviewer request, here we analyze the effect of demographic variables on the performance of Tsimane’ participants. Specifically, we conducted a mixed effects model predicting whether or not the participant’s response aligned with our hypothesis based on age, gender, and schooling with intercepts for study and participant (for Studies 5-7). We find that age and gender do not have a significant effect on the probability that participants responded in line with our hypotheses ($\beta_{age} = 0.004$, $p = 0.459$; $\beta_{gender} = -0.16$, $p = 0.260$), but years of schooling does have a small significant effect improving the probability that the participant will respond in line with our hypotheses ($\beta_{schooling} = 0.04$, $p = 0.03$). We believe this effect of education is consistent with related findings in the Tsimane’ showing that education yields an improvement on experimental tasks, possibly due to increased comfort in experimental tasks [7]. However, it is likely that years of schooling in the Tsimane’ correlate with other life experiences. Therefore, further work is needed to delve into this issue and as such our cross-cultural findings should be treated as only preliminary evidence of the effect of rarity and repetition on communicativeness judgements outside of a US context.

Video Norming (AsPredicted #17044)

Thirty participants rated the rarity of the natural punctuality video set (mean age = 33.83, range = 21-61), thirty participants rated the repetitiveness of the natural punctuality video set (mean age = 35.63, range = 21-65), thirty participants rated the rarity of the low punctuality video set (mean age = 38.07, range = 22-71), and thirty participants rated the repetitiveness of the low punctuality video set (mean age = 33.13, range = 20-68). All participants were recruited from Amazon Mechanical Turk through the TurkPrime platform.

The participants who rated the rarity of the videos were shown pairs of videos (the basic and the rare version of the same movement type from the same video set) and asked to indicate which action was more "uncommon," which we defined as any actions that seems unusual or like an action that you would not frequently see another person doing in your day to day life. Participants responded to the question "Which action is more uncommon?" on a Likert scale of one (*definitely the action in video 1*) to seven (*definitely the action in video 2*) with a median of 4 (*equally uncommon*). Participants were assigned to one of four trial orders in which the videos were counterbalanced for which version of the movement type was video 1 and video 2.

The procedure for participants rating the repetitiveness of the videos was identical to the procedure described for the rarity norming process except that participants were shown pairs of videos (the basic and the repetitive version of the same movement type from the same video set) and asked to indicate which action was more repetitive.

See Supplementary Table 6 for item averages and confidence intervals.

Supplementary Study based on Schachner & Carey (2013) Study 1

This study is a conceptual replication and extension of Study 1 in Schachner and Carey [8], which investigated inferences about movement-based goals. In the original study, participants watched an agent making repetitive inefficient movements, and they inferred that the goal of the movement was to produce the movement itself. At first, the lack of a communicative inference in this study appears to contradict our account.

We hypothesized that this phenomena was the result of Schachner and Carey using an event where communication was a priori implausible. In their stimuli, the agent was making inefficient and repetitive actions, but was alone and the eyes were always staring either to the side or towards the sky. Therefore, even if the movement looked communicative, participants may have rejected this interpretation due to the lack of a potential addressee and the agent's fixed attention to an empty region at the top or side of the screen.

Note that this hypothesis is not a challenge to Schachner and Carey (2013). Schachner and Carey (2013) concluded that people infer that the goal of a movement is the movement itself when no other interpretation is available, making it important that the movement could not be seen as communicative. If our account is correct, however, the presence of a potential recipient, and the removal of the averted gaze should lead people to interpret the movement as communicative. To test this, we modified the stimuli from Schachner & Carey ([8]; Study 1) so that the agent was shown from the back (therefore removing eye gaze information) and an observer was included in the scene. In line with Schachner & Carey (2013), we replicated the finding that participants explain the movement as world-directed when contextual information supported such interpretation (Objects Present condition; similar to the logic of Study 3 in our manuscript). In contrast to Schachner & Carey (2013), we found that participants now interpreted the movement as communicative then the world-directed contextual information was removed (Objects Absent condition). This shows that our work is consistent with Schachner & Carey (2013), and extends their results, showing that the movements in their study are treated as communicative with minimal changes to contextual information that had ruled out this possibility in their study.

Sample Size. Our sample size was 70 US participants recruited online through Prolific. In line with Schachner and Carey (2013), 50 participants were assigned to the Objects Absent condition and 20 participants completed the Objects Present condition.

Stimuli. Stimuli consisted of two short video animations based on the publicly available stimuli from the *Objects Present* and *Objects Absent* conditions from Study 1 of Schachner and Carey (2013). The videos were modified with Adobe After Effects in two ways, shown in Supplementary Fig. 11. First we removed the eyes from the main agent "Tim" in both videos. Second, we added in a second agent (a green circle with eyes) to serve as a potential communicative recipient. Additionally, we created a third video for use in the cover story, which had Tim (with two eyes) facing the participant on a white screen before turning 180 degrees, so that the eyes were no longer visible and the participants would understand that they were now looking at the back of Tim. This was done in order to establish that Tim was an agent with eyes, but that the participant would not be able to see his eyes all the time. The edited videos are available at our OSF site.

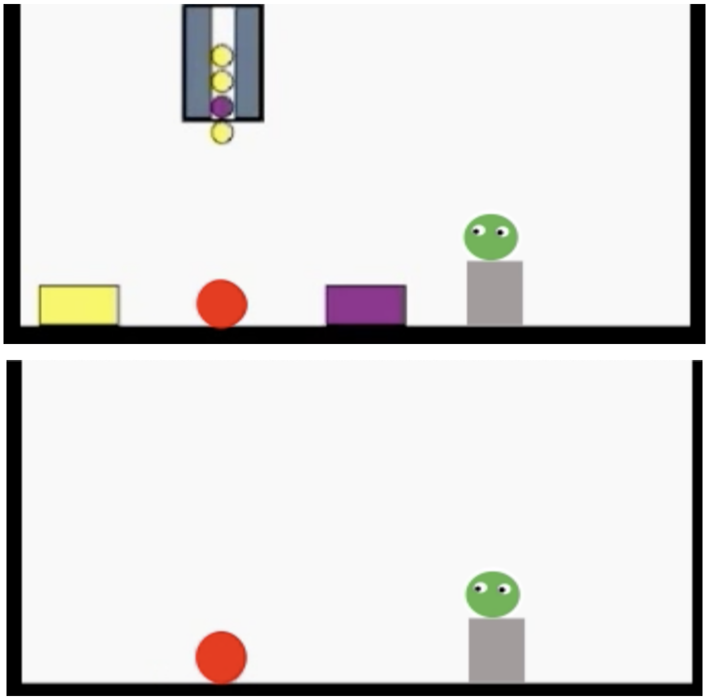

Supplementary Fig 11. Screenshots from the Objects Present condition and the Objects Absent condition of our supplementary study.

Cover Story. Participants read the following cover story: "*You are going to watch a short video featuring a character named Tim. This is Tim* [participants watched a short video of a red circle with eyes turning away from them so that they would understand that Tim is an agent with eyes, even if the eyes were not visible in the other stimuli]. *After watching a video, you will be asked to guess what was Tim's intention in the video. In other words, what was the goal of Tim's movements.*"

Procedure. After reading the cover story, participants saw either the Objects Present or Objects Absent video and had to respond to the question "In the video, what Tim's intention?"

The prompt, "In the video, what was Tim's intention?", was the same as the prompt used in Schachner and Carey (2013). However, participants in the original study were first asked, "What was Tim doing?" as an exclusion question and were later asked to predict future action (which we did not include since it was not relevant to our aims here).

Coding Scheme. Participant responses were categorized by two coders blind to the stimuli information. Coders categorized each response into one of five categories: *external goal*: the goal is some sort of physical means to an end. The goal is about something in the physical environment or meant to change the character's physical location in the environment;

communicative goal: The goal is to interact with another character or the participant; *movement-based goal*: The goal is simply to perform certain movements; *description:* The response describes low-level movements without giving any indication that the character's goal was to perform those movements*; and other*: the response is nonsensical or does not fit into any of the categories listed above. Any category disagreements were settled by a third blind coder who selected between the two categories chosen by the first two coders.

While we used Schachner and Carey's definitions for external and movement-based goals, unlike Schachner and Carey (2013), we added in an additional category for communicative goals to more easily assess the effects of including a potential observer. Additionally, Schachner and Carey's "ambiguous" and "no goal" categories were collapsed into our *other* condition. We also purposefully differentiated between movement-based goals, which stated that the agent intended to perform those movements (e.g., Tim intended to jump up and down) and descriptions of the movement, which recounted the movements without any reference to a goal or intention to move in that way (e.g., "Tim jumps, lands, moves left, then moves back to original spot. Tim jumps, lands, moves right, then moves back to the original spot. Tim jumps, lands, then moves left, then back to the original spot. Then it loops."). Since participants were asked about an intention, simply describing the movement without appealing to any motives does not constitute a valid answer to the critical question and may indicate that the participant was confused or could not articulate a goal for the movement.

Results and Discussion. In line with the findings of Schachner and Carey, most participants inferred external goals in the Objects Present condition (75% of participants in our study; Supplementary Fig. 12). However, in our study, 42% of participants in the Objects Absent condition inferred a communicative goal and only 8% inferred a movement-based goal. In the original study, 49% of participants in the Objects Absent condition inferred movement-based goals. The fact that the percentage of movement-based goals in Schachner & Carey's study was comparable to the percentage of communicative goals in our study suggests that people indeed believe the movements can be interpreted as communicative, but they rejected this explanation in the previous study due to the absence of a recipient, or due to the agent's eye-gaze.

These findings provide further evidence of the importance of contextual features when attributing different goals. In this case, a potential recipient may constitute a precondition for inferring communicative goals.

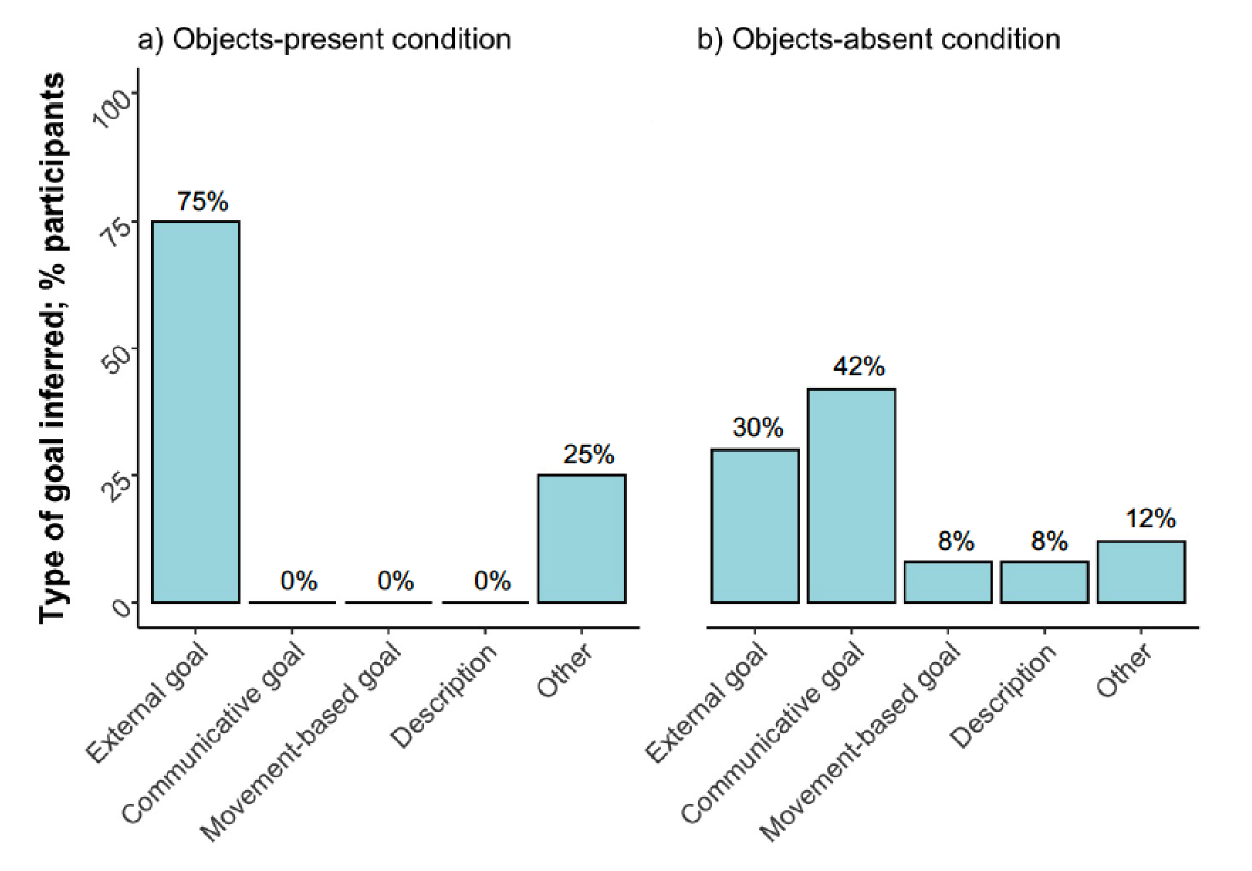

Supplementary Fig 12. Results from our follow-up study to Schachner & Carey (2013)'s Study 1 [8].

Six Layer Consent Process for Obtaining Consent from Tsimane' Participants

A critical aspect of research with the Tsimane' is sensitivity to their cultural context. For instance, whereas participants in the US understand that a statement like "we do not expect this procedure to cause any pain" is a legal disclosure, the Tsimane' often reasonably interpret those kinds of judgments as suggestive that there is a reasonable expectation for adverse outcomes. As such, "full" disclosure of information can sometimes lead to confusion rather than clarification.

All recruitment procedures are done as directed by the Grand Tsimane' Council, using a layered process of consent. The process of participant recruitment is described in terms of the layers of consent:

<u>First layer of consent:</u>

The Centro Boliviano de Investigación y Desarrollo Socio Integral (CBIDSI) allows the approved interpreters to broadcast a message to the community that the researchers are visiting. This broadcast is usually sent between one and three days in advance. In this broadcast, the Tsimane' are informed that researchers will be visiting, and that anyone who is interested in participating in the tasks can stay in the village (the Tsimane' often leave their villages during the day and return at nighttime). Tsimane' communities are visited often by researchers (to our knowledge, at least eight

different groups of researchers visit the communities at least once every other year). Listeners are reminded that staying in the community to participate in the studies is voluntary.

Second layer of consent:

When the researchers arrive, a town meeting is called, which happens in the village's community center. When everyone has arrived, the interpreters give a presentation where they tell members of the community the nature of the experiment that is being run, how long the task is, and the compensation for participating. After the presentation, a conversation in Tsimane' usually follows where the members of the community openly ask questions to the interpreters. It is important to note that the Tsimane' are accustomed to the research we conduct and the questions at this point usually focus on procedural questions (e.g. how many compensation bags do the experimenters have? If they run out, can we still participate and have the experimenters bring the compensation the next day?). This entire procedure is done as directed by the Grand Tsimane' Council.

Third layer of consent:

Once the meeting has ended, members of the community are reminded that the task is voluntary and they are asked to come forward if they wish to participate. A list of members who express interest in participating is recorded (see section below on collecting information about individuals).

Fourth layer of consent:

When the participant approaches the table where the experiment is run, the interpreter confirms that the member was present during the town meeting. If they were, the interpreter asks if they wish to participate, and they are reminded that this is voluntary and that they may leave at any point. If the participant was not at the town meeting, the interpreter explains everything that was covered during the meeting. Once the participant is up-to-date, they are asked if they wish to participate.

Fifth layer of consent:

As a fifth layer of consent, we request the interpreter (who has been previously familiarized with the consent form) to read the consent form determined by the IRB. At this point, we explain to participants that this is a requirement from an ethics committee in the US. All information in the form has already been repeated in the first four layers. If the interpreter is unable to read, the experimenter reminds the interpreter of each point in the assent form sequentially. It should be noted that the Grand Tsimane' Council does not consider this stage necessary, as they deem the rest of the layers of consent more important and appropriate. However, they recognize that this may be required by US university regulations.

Sixth layer of consent:

Finally, the experimenters explain that we would like to video tape or audio tape the experiment in order to keep a record of the data that we collected, and video/audio is only recorded when the participant approves.

Vocal Communication and the Pressure to Reveal Communicative Goals

Our work focused on the recognition of physical communicative action, including an analysis of how our framework applies to sign languages. But of course, spoken languages are often people's primary form of communication. Here, we consider whether pressures to reveal a communicative goal apply to speech-based communication as well.

Vocalizations can be broadly thought of as falling into three categories: non-communicative non-speech vocalizations (e.g., coughing, yawning), communicative non-speech vocalizations (e.g., gasps, disgust vocalizations, but also vocalizations that are typically non-communicative, but can be used with a communicative intent, such as coughing to get someone's attention, or yawning to intentionally communicate boredom), and speech (e.g., words and sentences). Therefore, if we apply our framework to the domain of vocal communication, then this would generate the hypothesis that people expect communicative vocalizations to reveal that they are not non-communicative vocalizations. We begin by considering this hypothesis in the case of speech, followed by the case of communicative non-speech vocalizations.

Identifying speech. There are two broad reasons why people may not need to expect speech to disambiguate itself from non-speech vocalizations.

The first reason is parallel to the reason why sign languages may not require every sign to disambiguate itself from world-directed goals (see Discussion in main text): Speech—much like signing—most often occurs in extended communicative interactions. This means that, as soon as a sound is recognized as speech, the listener can continue to expect the following sounds to also be speech until an utterance has been completed. Moreover, speech occurs in contexts where agents engage in turn-taking interactions with ostensive cues that are salient to even infants [*9-12*]. This context likely helps listeners assume that the sounds are communicative and may reduce the burden on the sound itself to reveal its communicative goal. Indeed, the effects of a communicative context on the interpretation of sound are so strong that it can even lead infants to treat highly artificial non-biological sounds as communicative. In one recent study, infants learned abstract patterns from sine-wave tones (a phenomenon previously proposed to be specific speech-based communication) when the tones were dubbed over a communicative interaction, but not when the same tones were presented in a noncommunicative context [*13*].

The second reason why people may not need to expect speech to disambiguate itself from non-speech vocalizations is because this disambiguation might happen at a perceptual, rather than cognitive, level. Speech exhibits highly efficient neural encoding such that the greatest amount of auditory information is captured with the lowest neural effort [see *14* for review]. Similarly, infants prefer to listen to speech over non-speech stimuli (both when contrasted with acoustically similar artificial sounds and non-speech vocalizations such as gasps or disgust sounds) even when critical features of the stimuli are held constant [*15,16*]. As such, it has long been argued that speech has evolved a "special" status in comparison to other types of auditory input [*17,18*].

At an even broader level, it is possible that speech evolved as a primary medium of communication because those sounds were not historically used to perform non-communicative world-directed actions. Therefore this would make those sounds an ideal channel where listeners do not face high-levels of ambiguity when speech is produced. Note however, that there is significant debate about how and why the modality of linguistic communication evolved as it did [see *19* for review].

Identifying communicative vocalizations outside speech. Beyond speech, people often communicate through non-speech vocalizations, such as yawning or coughing. Sometimes these sounds fulfill biological functions and sometimes these sounds are used to communicate (e.g., to convey boredom or indicate that a faux pas has occurred). Moreover, these sounds are often used to communicate outside the context of an ongoing communicative interaction, so the contextual cues that help observers to treat linguistic stimuli as communicative are likely insufficient. These situations therefore impose inferential demands on listeners (is this vocalization communicative or just a non-communicative biological vocalization?), which parallel the ones observers face in the case of communicative action (is this movement communicative or just world-directed?). In this way, these communicative non-speech vocalizations parallel the characteristics of emblems (see main text). We therefore predict that our framework's abstract principles apply here. In these cases, listeners may expect communicative non-speech vocalizations to reveal that they are indeed communicative. Critically, this does not imply that listeners should expect these sounds to be rare or repetitive, as these strategies were derived to reveal that a movement is not world-directed. Instead, our framework would predict that communicators should find ways to modulate the sound in a way that allows listeners to recognize that the vocalization is not a typical non-communicative vocalization.

Similarly, we also do not predict that repetition or other features would be built into complete spoken languages in order to differentiate speech from non-communicative vocalizations. While repetition is a notable form of non-arbitrariness in both signed and spoken languages, its purpose is not to signal that speech is communicative, but rather it is often used iconically to connect lexical items to their meanings [*20*]. However, repeated elements (i.e., reduplication) are common in baby talk (e.g., night-night and choo-choo) and these reduplicated words are easier for infants to learn [*21,22*]. It has been proposed that these reduplicated baby talk words "...may be more likely to be noticed in the input and stored in verbal memory than their adult-like counterparts (e.g., *train* and *good night*), making them accessible targets for initial word learning" [*23*, p.1979]. Why reduplicated elements have an early learning advantage is still an open question that could relate to the identifiability of reduplicated baby talk words as discrete communicative signals.

Gesture Regression Tables

Supplementary Table 1: Video Norming

**Movement Type**	**Rarity**<br>**Low Punctuality**	**Rarity**<br>**High Punctuality**	**Repetition**<br>**Low Punctuality**	**Repetition**<br>**High Punctuality**
Chin Swosh	2.44<br>(1.89, 3.05)	2.74<br>(2.19, 3.32)	2.58<br>(1.94, 3.26)	2.74<br>(2.07, 3.47)
Horizontal Swosh	2.47<br>(1.88, 3.14)	2.39<br>(1.85, 3.02)	2.27<br>(1.68, 2.90)	2.05<br>(1.57, 2.61)
Cheek Brush	2.20<br>(1.71, 2.77)	3.08<br>(2.53, 3.68)	2.40<br>(1.82, 3.03)	2.11<br>(1.61, 2.65)
Hair Touch	1.89<br>(1.43, 2.43)	2.19<br>(1.68, 2.80)	2.28<br>(1.76, 2.86)	2.02<br>(1.54, 2.57)
Vertical Swosh	2.11<br>(1.67, 2.58)	2.01<br>(1.56, 2.53)	2.63<br>(1.96, 3.38)	2.38<br>(1.81, 3.01)
Finger Swosh	1.87<br>(1.44, 2.39)	1.90<br>(1.56, 2.29)	2.14<br>(1.65, 2.71)	2.11<br>(1.64, 2.62)

Pre-registration Divergences

Study 1: No pre-registration.
Study 1 Replication (#12593): In addition to our pre-registered logistic mixed effects regression, we also conducted a Pearson's correlation test.
Study 2: No pre-registration.
Study 2 Replication (#13925): None. Note: Title of pre-registration refers to experiment as "Study 3."
Study 3: No pre-registration.
Study 3 Replication (#13923): None. Note: Title of pre-registration refers to experiment as "Study 2."
Explanation Control: No pre-registration.
Explanation Control Replication (#13961): None. Note: Title of pre-registration refers to experiment as "Study 4."
Study 4 (#12589, #12590): In addition to our pre-registered analyses, we also conducted a mixed effects model predicting participants' communicativeness judgments based on the demonstrator in the video.
Weirdness Control: None.
Familiarity Control (#12589, #12590): Instead of separately analyzing data from the study with the low punctuality video set (referred to in pre-registrations as the "knowledgeable demonstrator") and the natural punctuality video set (naive demonstrator), we combined the data for this analysis because communicativeness judgments in Study 4 were not significantly predicted by the demonstrator.
Study 5 (#17032, #13317, #13223, #13388): In order to compare across populations, we use a mixed effects model.
Study 6 (#13960): We pre-registered that we would collect data till we achieve a sample size of 60 participants giving variance in their responses. However, after finishing data collection, we realized that one participant was mislabeled as having provided variance in her responses. So our final sample size only includes 59 participants.
Study 7 (#26450): Because we excluded responses categorized as "other" and "descriptive" leaving us with unequal numbers of video types, we could not conduct the pre-registered bootstrap of the average difference between participant responses for different video types. Instead, we only conducted the pre-registered mixed effects model.

Supplementary References

[1] Chris L Baker, Rebecca Saxe, and Joshua B Tenenbaum. "Action understanding as inverse planning". In: *Cognition* 113.3 (2009), pp. 329–349.

[2] Chris L Baker et al. "Rational quantitative attribution of beliefs, desires and percepts in human mentalizing". In: *Nature Human Behaviour* 1.4 (2017), p. 0064.

[3] Julian Jara-Ettinger, Chris Baker, and Joshua Tenenbaum. "Learning what is where from social observations". In: *Proceedings of the Annual Meeting of the Cognitive Science Society*. Vol. 34. 34. 2012.

[4] Julian Jara-Ettinger, Laura E Schulz, and Joshua B Tenenbaum. "A Naive Utility Calculus as the foundation of action understanding". In: (under review).

[5] Richard S Sutton and Andrew G Barto. *Reinforcement learning: An introduction*. MIT press, 2018.

[6] Joey Velez-Ginorio et al. "Interpreting actions by attributing compositional desires.(2017)". In: (2017).

[7] Edward Gibson, Julian Jara-Ettinger, Roger Levy, and Steven Piantadosi. (2017). The use of a computer display exaggerates the connection between education and approximate number ability in remote populations. Open Mind, 2(1), 37-46.

[8] Adena Schachner and Susan Carey (2013). Reasoning about 'irrational' actions: When intentional movements cannot be explained, the movements themselves are seen as the goal. Cognition, 129(2), 309-327.

[9] Csibra, G. (2010). Recognizing communicative intentions in infancy. *Mind & Language*, *25*(2), 141-168.

[10] Csibra, G., & Gergely, G. (2009). Natural pedagogy. *Trends in cognitive sciences*, *13*(4), 148-153.

[11] Esseily, R., & Fagard, J. (2013). Ostensive cues orient 10-month-olds' attention toward the task but delay learning. *Psychology*, *4*(07), 20.

[12] Senju, A., & Csibra, G. (2008). Gaze following in human infants depends on communicative signals. *Current biology*, *18*(9), 668-671.

[13] Ferguson, B., & Lew-Williams, C. (2016). Communicative signals support abstract rule learning by 7-month-old infants. *Scientific reports*, *6*(1), 1-7

[14] Gervain, J., & Geffen, M. N. (2019). Efficient neural coding in auditory and speech perception. *Trends in neurosciences*, *42*(1), 56-65.

[15] Vouloumanos, A., & Werker, J. F. (2007). Listening to language at birth: Evidence for a bias for speech in neonates. *Developmental science*, *10*(2), 159-164.

[16] Shultz, S., & Vouloumanos, A. (2010). Three-month-olds prefer speech to other naturally occurring signals. *Language Learning and Development*, *6*(4), 241-257.

[17] Liberman, A. M. (1984). On finding that speech is special. In *Handbook of Cognitive Neuroscience* (pp. 169-197). Springer, Boston, MA.

[18] Vatakis, A., Ghazanfar, A. A., & Spence, C. (2008). Facilitation of multisensory integration by the "unity effect" reveals that speech is special. *Journal of Vision*, *8*(9), 14-14.

[19] Zywiczynski, P., Gontier, N., & Wacewicz, S. (2017). The evolution of (proto-) language: Focus on mechanisms.

[20] Dingemanse, M., Blasi, D. E., Lupyan, G., Christiansen, M. H., & Monaghan, P. (2015). Arbitrariness, iconicity, and systematicity in language. *Trends in cognitive sciences*, *19*(10), 603-615.

[21] Gervain, J., Macagno, F., Cogoi, S., Peña, M., & Mehler, J. (2008). The neonate brain detects speech structure. *Proceedings of the National Academy of Sciences*, *105*(37), 14222-14227.

[22] Ota, M., & Skarabela, B. (2016). Reduplicated words are easier to learn. *Language Learning and Development*, *12*(4), 380-397.

[23] Ota, M., Davies‐Jenkins, N., & Skarabela, B. (2018). Why Choo‐Choo Is Better Than Train: The Role of Register‐Specific Words in Early Vocabulary Growth. *Cognitive Science*, *42*(6), 1974-1999.
