# Supplementary material for: People infer communicative action through an expectation for efficient communication
Source: Nat Commun. 2022 Jul 18;13:4160. doi: 10.1038/s41467-022-31716-3 (PMC9293910; doi:10.1038/s41467-022-31716-3)
Supplement: Supplementary file 3 — Reporting Summary [file 41467_2022_31716_MOESM3_ESM.pdf]

nature research

Corresponding author(s): Amanda Royka

Last updated by author(s): May 29, 2022

Reporting Summary

Nature Research wishes to improve the reproducibility of the work that we publish. This form provides structure for consistency and transparency in reporting. For further information on Nature Research policies, see our Editorial Policies and the Editorial Policy Checklist.

Statistics

For all statistical analyses, confirm that the following items are present in the figure legend, table legend, main text, or Methods section.

n/a	Confirmed	
☐	☒	The exact sample size (*n*) for each experimental group/condition, given as a discrete number and unit of measurement
☐	☒	A statement on whether measurements were taken from distinct samples or whether the same sample was measured repeatedly
☐	☒	The statistical test(s) used AND whether they are one- or two-sided<br>*Only common tests should be described solely by name; describe more complex techniques in the Methods section.*
☐	☒	A description of all covariates tested
☐	☒	A description of any assumptions or corrections, such as tests of normality and adjustment for multiple comparisons
☐	☒	A full description of the statistical parameters including central tendency (e.g. means) or other basic estimates (e.g. regression coefficient) AND variation (e.g. standard deviation) or associated estimates of uncertainty (e.g. confidence intervals)
☐	☒	For null hypothesis testing, the test statistic (e.g. *F*, *t*, *r*) with confidence intervals, effect sizes, degrees of freedom and *P* value noted<br>*Give P values as exact values whenever suitable.*
☐	☒	For Bayesian analysis, information on the choice of priors and Markov chain Monte Carlo settings
☐	☒	For hierarchical and complex designs, identification of the appropriate level for tests and full reporting of outcomes
☐	☒	Estimates of effect sizes (e.g. Cohen's *d*, Pearson's *r*), indicating how they were calculated

Our web collection on statistics for biologists contains articles on many of the points above.

Software and code

Policy information about availability of computer code

Data collection	Qualtrics online survey platform, Amazon Mechanical Turk, and Turk Prime were all used in the process of data collection. Prolific was used to collect data for the supplemental study based on Schachner & Carey, 2013.
Data analysis	R Studio (Version 1.2.5042) and R packages lmerTest (3.1-2), tidyverse (1.3.0), boot (3.1-25), and stargazer (5.2.2) were used to analyze our data. Additionally, we provide our own data analysis R code on the OSF pages (https://osf.io/cnf5h/ and https://osf.io/sw2un/) referenced in our data availability statement.

For manuscripts utilizing custom algorithms or software that are central to the research but not yet described in published literature, software must be made available to editors and reviewers. We strongly encourage code deposition in a community repository (e.g. GitHub). See the Nature Research guidelines for submitting code & software for further information.

Data

Policy information about availability of data

All manuscripts must include a data availability statement. This statement should provide the following information, where applicable:

- Accession codes, unique identifiers, or web links for publicly available datasets
- A list of figures that have associated raw data
- A description of any restrictions on data availability

All associated pre-registrations, stimuli, and datasets are publicly available on the Open Science Framework. Files from Studies 1-3 and the Explanation Control are available at https://osf.io/ehb48/. Files from Studies 4-7 are available at https://osf.io/wxdka/. Figures 2,3, 5, 6, and 7 were created using data from their corresponding studies as indicated in the figure captions.

April 2020

Field-specific reporting

Please select the one below that is the best fit for your research. If you are not sure, read the appropriate sections before making your selection.

☐ Life sciences ☒ Behavioural & social sciences ☐ Ecological, evolutionary & environmental sciences

For a reference copy of the document with all sections, see nature.com/documents/nr-reporting-summary-flat.pdf

Behavioural & social sciences study design

All studies must disclose on these points even when the disclosure is negative.

Study description	We present 8 studies based on 20 quantitative datasets with an additional supplementary study included to respond to a reviewer comment. We employ several different paradigms, including graded acceptability tasks, forced-choice tasks, indirect prompts, and open-ended explanation tasks, in both market-integrated and non-integrated communities.
Research sample	Amazon Mechanical Turkers were utilized for most of the studies reported in the manuscript. US participants (as indicated by their IP addresses) were recruited through Amazon's Mechanical Turk platform in exchange for monetary compensation based on the duration of the task. Samples of 30 participants were recruited for Study 1 (mean age = 32.87, range = 22-63), Study 1 replication (mean age = 35.40, range = 24-59), Study 2 (mean age = 34.23, range = 23-59), Study 2 replication (mean age = 36.40, range = 21-61), Study 3 (mean age = 40.83, range = 25-73), Study 3 replication (mean age = 37.80, range = 23-69), Explanation Control (mean age = 36.80, range = 21-63), and Explanation Control replication (mean age = 35.93, range = 23-50). An additional 150 US participants were recruited through AMT: 30 participants each completed each Study 4 low punctuality (mean age = 38.40, range = 23-60); Study 4 natural punctuality (mean age = 34.17, range = 23-58); Weirdness Control (mean age = 37.73, range = 23-60); low punctuality Familiarity Control (mean age = 36.8, range = 22-68); and natural punctuality Familiarity Control (mean age = 35.70, range = 20-60). For Study 5, 40 San Borjan participants (mean age = 31.78, range = 16-63, 26 female participants) were recruited in San Borja, Bolivia near the town square, and 180 Tsimane' participants (mean age = 31.97, range = 14-71, 122 female participants) were recruited in their communities, and 40 US participants were recruited from the Yale University undergraduate subject pool. 59 US participants (mean age = 36.39, range = 23-71) were recruited through AMT and 59 Tsimane' participants (mean age = 30.86, range = 15-64, 35 female participants) were recruited in their local communities. 100 US participants (mean age = 41.52, range = 20-77) were recruited through Amazon's Mechanical Turk platform and 32 Tsimane' participants (mean age = 34.88, range = 19-73, 24 female participants) were recruited from their local communities. For AMT and Yale participants, gender information was not recorded, but AMT's gender demographics were 55% female for US participants in 2017 when data collection began. Gender information for Bolivia participants was recorded for an unrelated project that was happening simultaneously and is thus reported here. None of our samples were representative.
Sampling strategy	All participants were recruited through random sampling. Sample size for studies 1-4 was based on related research on action understanding (n=30 per study; Jara-Ettinger, Schulz, & Tenenbaum, under review). Replications for studies 1-4 matched the sample size, as the original four studies revealed adequate power. We expected comparable effects in studies 5-8 and we thus aimed to collect 30 data points per stimuli. This led to different sample sizes depending on the number of stimuli and experimental design of each study (as some combinations of stimuli could not be presented within participants, as described in the main text).
Data collection	For studies using participants from Amazon Mechanical Turk, participants completed the studies on their own personal computing devices. Yale undergraduates, San Borjans, and Tsimane' were all run on the experiments on an iPad by an unblinded experimenter. Tsimane' were tested with an experimenter and a translator.
Timing	Study 1: 8/3/2017 - 8/9/2017; Study 2: 12/28/2017 - 1/2/2018, Study 3: 10/5/2017 - 10/21/2017; Explanation Control: 2/8/18 - 2/18/18; Study 1 rep: 9/5/18 - 9/12/18; Study 2 rep: 9/29/18 - 10/18/18 ; Study 3 rep: 9/11/18 - 9/21/18; Explanation Control rep: 11/9/18 - 11/21/18; Study 4: 7/17/18 - 7/24/18 (Low Punctuality), 7/9/18 - 7/22/18 (Natural Punctuality); Study 4 Control: 7/19/18 - 9/6/18 (Low Punctuality), 8/1/18 - 8/8/18 (Natural Punctuality); Study 5: 11/6/18 - 12/4/18 (Yale), 8/13/18 - 8/15/18 (San Borja), 8/8/18 - 8/16/18 (Tsimane' Low Punctuality), 8/16/18 - 8/17/18 (Tsimane' Natural Punctuality); Study 6: 8/21/18 - 8/23/18 (Tsimane'), 10/19/19 - 10/31/19 (MTurk); Study 7: 8/23/18 - 8/23/18 (Tsimane'), 8/6/19 - 8/21/19 (MTurk); Supplementary Study based on Schachner & Carey, 2013: 6/25/2021 - 6/28/2021
Data exclusions	For Study 6 (low punctuality demonstrator) 49 Tsimane' participants were recruited, but excluded from the study: 27 participants failed to correctly answer the warm-up questions and 22 participants were excluded because decided excluded all data from participants from a specific Tsimane' community because during testing we had reason to believe that participants had discussed the study, which caused later participants to point to videos before being asked questions. 9 Tsimane' participants were recruited, but excluded from a version of Study 6 using the natural punctuality video set; all said participants were excluded for failing the warm-up trials. Exclusions based on warm-up trials were pre-registered. For Study 7, 5 US participants were recruited but excluded because they gave the same response for all stimuli and 26 Tsimane' participants were recruited but excluded because they gave the same response for all stimuli. This exclusion criteria was pre-registered. For Study 8, one Tsimane' participant was excluded due to experimenter error.
Non-participation	No participants declined participation.
Randomization	For the amazon mechanical turk studies, participants were either random allocated into pre-determined trial orders or trials order was randomized (depending on the experimental design; all randomization are specified clearly in the Methods). For studies conducted in-person (with the Tsimane', San Borjans, and Yale undergraduates), participants were assigned to trial orders based on the order of participation. For example, if a study had 3 trial orders, the first participant saw trial order #1, the second saw trial order #2, the third trial order #3, the fourth trial order #1 and so on.

Reporting for specific materials, systems and methods

We require information from authors about some types of materials, experimental systems and methods used in many studies. Here, indicate whether each material, system or method listed is relevant to your study. If you are not sure if a list item applies to your research, read the appropriate section before selecting a response.

Materials & experimental systems

n/a	Involved in the study
☒	☐ Antibodies
☒	☐ Eukaryotic cell lines
☒	☐ Palaeontology and archaeology
☒	☐ Animals and other organisms
☐	☒ Human research participants
☒	☐ Clinical data
☒	☐ Dual use research of concern

Methods

n/a	Involved in the study
☒	☐ ChIP-seq
☒	☐ Flow cytometry
☒	☐ MRI-based neuroimaging

Human research participants

Policy information about studies involving human research participants

Population characteristics	See above.
Recruitment	Yale undergraduate participants were recruited through the University's study participant pool. Since these participants were undergraduates they reflect a biased age sample. Use of Amazon mechanical Turk helps to circumvent these biased demographic concerns to an extent since the participant pool is more age-diverse. All MTurk studies were posted to the platform under the "Survey" tag. Participants from San Borja were recruited near the town square. They were asked whether they would like to participate in a short survey in exchange for monetary compensation. The participation of the Tsimane' was coordinated through Centro Boliviano de Investigación y Desarrollo Socio Integral. While we do not anticipate that self-selection bias would impact our results since our theory posits that the recognition of novel communicative movements is rooted in basic goal-inferential processes, there was nonetheless the opportunity for self-selection bias to occur.
Ethics oversight	All studies detailed here received ethical approval from the Yale Human Subjects Committee and complied with all relevant ethical regulations. All studies conducted with the Tsimane’ also received local approval from the Grand Tsimane’ Council. Informed consent was obtained from all participants.

Note that full information on the approval of the study protocol must also be provided in the manuscript.
